# Anti-inflammatory potential of dichloromethane leaf extracts of *Eucalyptus globulus* (Labill) and *Senna didymobotrya* (Fresenius) in mice

**DOI:** 10.4314/ahs.v21i1.50

**Published:** 2021-03

**Authors:** Joseph Kiambi Mworia, Cromwell Mwiti Kibiti, Joseph JN Ngeranwa, Mathew Piero Ngugi

**Affiliations:** 1 Department of Biochemistry, Microbiology and Biotechnology, Kenyatta University, P.O Box 43844-00100, Nairobi, Kenya; 2 Department of Pure and Applied Sciences, Technical University of Mombasa, P.O Box 90420-80100, Mombasa, Kenya

**Keywords:** *Eucalyptus globulus*, *Senna didymobtrya*, inflammation, phytochemicals

## Abstract

**Background:**

Inflammation is an immune response characterized by swelling, redness, pain and heat. Inflammation is mainly managed using conventional medicines that are associated with many side effects. Plant-based remedies are considerably better alternative therapies for they have fewer side effects.

**Objective:**

This study aimed at determining the anti-inflammatory potential of dichloromethane (DCM) leaf extracts of *Eucalyptus globulus* and *Senna didymobotrya* in mice.

**Methods:**

Fresh leaves of these plants were harvested from Embu County, Kenya. Quantitative phytochemical analysis was done using Gas Chromatography-Mass Spectrometry (GC-MS). Anti-inflammatory test comprised nine groups of five animals each: normal, negative, positive controls and 6 experimental groups. Inflammation was induced with Carrageenan. One hour post-treatment, the different groups were intraperitoneally administered with the reference drug, diclofenac, 3% DMSO and six DCM leaf extracts at doses of 25, 50, 100, 150, 200 and 250mg/kgbw.

**Results:**

GC-MS results revealed α-phellandrene, camphene, terpinolene, and limonene among others. Anti-inflammatory effects showed that extract doses of 100,150,200 and 250mg/kg bw significantly reduced the inflamed paw. Doses of 200 and 250mg/kgbw in both plants were more potent and compared with diclofenac. *E. globulus* extract dose of 250mg kg bw reduced inflamed paw in the 1^st^, 2^nd^, 3^rd^ and 4^th^ hours, by 2.27,6.52,9.09 and 10.90% respectively while *S.didymobotrya* at similar dose ranges, inflamed paw reduced by 2.41, 5.43, 8.31 and 9.05% respectively.

**Conclusion:**

*E. globulus* and *S. didymobotrya* have potent anti-inflammatory activities, attributed to their constituent phytochemicals. This study confirms the traditional use of these plants in treating inflammation.

## Introduction

Inflammation is a complex biological reaction of the vascular tissues to noxious stimuli like damaged cells, irritants and pathogens^1^. The objective of the inflammatory response is to limit and remove the causative agents of inflammation as well as to initiate the healing process in the damaged cells^2^. Inflammation is mainly managed using NSAIDs such as Ibuprofen, Naproxen Sodium and Diclofenac among others^3^. These drugs are associated with numerous side effects in the gastrointestinal tract, kidney, liver, cardiovascular system among others 4. Inflammation is also managed using medicinal plants such as *Azima tetracantha* (Lam) 5, *Eugenia jambolana*^6^ among others. Herbal medicines serve as better alternatives for they are arguably readily available, cheap and possess fewer side effects hence the need to search for new bioactive compounds in medicinal plants 7. *E. globulus* is an evergreen tree with a straight trunk of about 0.6 to 2 meters in diameter and a height of 40–70 meters tall. It has well spread deep roots along with smooth, brownish, greenish, mottled gray and long peeling bark. Most of them grow naturally in many parts of Kenya^8^. *E. globulus* is used in the management of abscess, asthma, burns, sore throat, bronchitis, colds, among others^9^. *S. didymobotrya* was introduced as an ornamental plant and grow in almost every part of Kenya. It is a bushy shrub found at altitudes of between 1450–2400 meters above the sea level. The plant grows to a maximum height of about 30–90cm^10^. It is used in the management of fungal, bacterial, parasitic infections, hypertension among others^11^. In Embu County, Kenya, *E. globulus* and *S. didymobotrya* are used in the traditional medicine in treatment of inflammation. However, there is no scientific data to confirm and/or validate this use. This study sought to explore the anti-inflammatory potential of dichloromethane leaf extracts of *E. globulus* (Labill) and *S. didymobotrya* (Fresenius) in mice models.

## Materials and methods

### Plant samples harvesting, processing, and extraction

The researchers obtained research authorization to undertake this study from National Commission for Science, Technology and Innovation, Kenya (NACOSTI/P/16/6765/1452). The collection of the plant samples was carried out based on ethnobotanical information availed by a local herbalist in Mbeere North Subcounty, Embu County, Kenya. Fresh leaves of *E. globulus* (Labill) and *S. didymobotrya* (Fresenius) were collected at GPS location of 0o35′12.′S, 37o38′32′E and 0o35′28.′S, 37o38′25′E respectively. The samples were cleaned, enveloped in Khaki bags and carried to Kenyatta University in the Department of Biochemistry, Microbiology and Biotechnology, where the study was undertaken. The samples were identified by Mr. Stephen Mwangi, a taxonomist in Kenyatta University. Voucher specimens of *E. globulus* (Labill) and *S. didymobotrya* (Fresenius) were deposited at the Plant Sciences Herbarium of Kenyatta University and were assigned voucher specimen numbers JKM001 and JKM002, respectively.

The samples were air-dried at 25ºC in the plant drying facility of Kenyatta University for 14 days. The dried samples were then milled separately into fine powders using an electric mill. The powders were stored at room temperature in well-sealed and labeled khaki papers awaiting extraction^12^.

For extraction, six hundred grams of each plant powder were separately weighed and put into well-labeled conical flasks. Two liters of dichloromethane were added into each conical flask, corked and left to stand for 24 hours with regular swirling every 6 hours. The mixtures were then decanted in clean and well labeled conical flasks. Filtration was then done using Whatman No.1 filter papers. Each filtrate was put in a well-labeled conical flask. One liter of dichloromethane was added to each residue and then left to stand for 24 hours, followed by decantation and filtration. This procedure was repeated until dichloromethane remained clear. Each extract was concentrated using a rotary evaporator at 40ºC until dry. The semi-solid extract was separately put in an open clean beaker for 5 days to evaporate the remaining solvent. A sticky solid was obtained and store stored at -4°C until use^13^.

### GC-MS analysis

**Figure 1:**
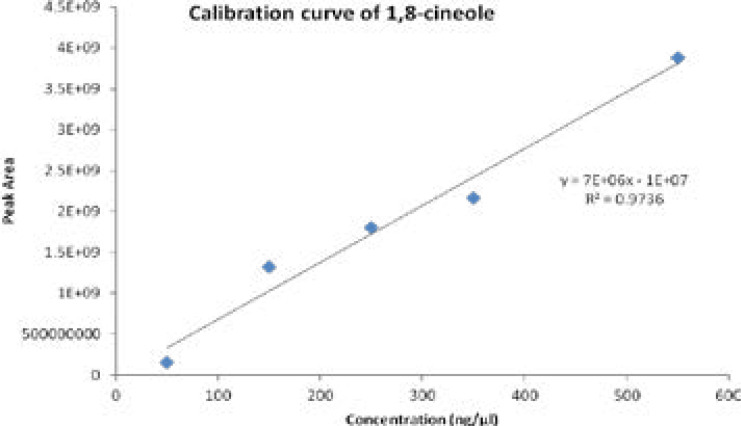
The calibration curve of 1,8-cineole (peak area vs. concentration)

Quantitative phytochemical analysis of the extract was done at the International Centre of Insect Physiology and Ecology (ICIPE) laboratories. A protocol reviewed by the Principal Scientist and Head of the Department of Behavioral and Chemical Ecology at ICIPE, Prof. Baldwyn Torto, was used to analyze the two plant extracts. The DCM extracts of *E. globulus* (Labill) and *S. didymobotrya* (Fresenius) DCM weighing 1.1 and 1.2mg respectively were diluted in their respective volumes through partitionng between hexane and methanol. This was followed by vortexes and centrifugation. Bypassing through anhydrous Na_2_SO_4_, the hexane layer was dried and then analyzed using GC-MS. Standard authentic serial dilutions (1,8-cineole; 99%, Gillingham, Dorset, England) (50ng/µl, 150ng/µl, 250ng/µl, 350 ng/µl and 550 ng/µl) were prepared, analyzed using GC-MS and their peaks used in quantification. The calibration curve of 1,8-cineole (peak area vs. concentration), with the equation y=7E+06-1E+07, served as the basis for the external quantification of the target compounds Figure 1.

Agilent Gas Chromatograph 7890A/5975C coupled to Mass Spectrometer in full scan mode was used to analyze the two plant extracts using the following specifications; gas chromatography Column (HP-5 MS low bleed capillary column (0.25µm, 30 m by 0.25 mm i.d.) (J and W, Folsom, California, United States of America), flow rate, (Helium) (constant flow mode at 1.25 ml/min), injection split mode, the temperature of the oven (35°C) for initial 5 minutes and then raised by 10°C per minute to 280°C for 10.5 minutes; run time 70 minutes^14^. The percentage abundance of each phytochemical identified by GC-MS was computed using the formula below;

### Experimental animals

Swiss albino mice weighing between 20 to 25 grams and aged 5–6 weeks were used in the present study. They were obtained from Kenyatta University Animal Breeding Facility. The mice were provided with laboratory diet, which comprised rodent pellets and *water ad libitum*. The mice were housed in groups of nine in standard polypropylene cages and maintained at room temperature (23 ± 2°C), bench level lighting of 360 lux and humidity (55 ± 5%) with 12hrs light and 12hrs dark cycle. Approval for use of laboratory animals in this study was obtained from the Ethics Committee for the Care and Use of Laboratory Animals of Kenyatta University, Kenya (KU/AERC/107-012/2017). Development of the experimental protocols and procedures were performed under the guidance of the Veterinarian, who is a member of the Kenya Veterinary Board (KVB). All procedures were carried out following the Public Health Service (PHS) Policy on Humane Care and Use Committee (IACUC) (Section 8.3.2) and KVB.

### Induction of inflammation

To induce inflammation, each mouse was injected with 0.1ml of 1% Carrageenan (commercial grade type 1) solution in normal saline in the left hind paw. Induction of inflammation was indicated by the manifestation of edema on the hind paw^15^.

### Experimental design

A completely randomized controlled study design was adopted in this study from which an experimental design was derived. Swiss albino mice were categorized into 9 sets of 5 mice each. The animals received treatments as follows; Group I (normal control) mice received 3% DMSO only and were not induced with inflammation^16^. Group II (negative control) mice were induced with inflammation and administered with 3% DMSO after one hour^17^. Group III (positive control) animals were induced with inflammation and then treated with the reference drug diclofenac sodium at a dose of 15mg/kg body weight after one hour^18^. Animals in groups (IV, V, VI, VII, VIII, and IX) were induced with inflammation and then treated with extracts dosages of 25, 50, 100, 150, 200 and 250mg/kg bw respectively after one hour. All the treatment doses were freshly prepared. The summary of this design is detailed in Table 1. The paw circumference (edema) was determined one hour post induction of inflammation and at hourly intervals after administration of different treatments for four hours. The paw circumference was measured using cotton thread and then transferred to the ruler to obtain the reading in millimeters^19^. The percentage edema inhibition was computed using the formula described by Umamageswari and Kudagi (2015)^20^.

**Table 1:** Summary of experimental design for determination of anti-inflammatory activities of dichloromethane leaf extracts of *Eucalyptus globulus* (Labill) and *Senna didymobotrya* (Fresenius) extracts on carrageenan-induced edema in Swiss albino mice

Group	Treatment
I	DMSO only
II	Carrageenan + DMSO
III	Carrageenan + diclofenac (15mg/kg body weight)
IV	Carrageenan + extract (25mg/kg body weight)
V	Carrageenan + extract (50mg/kg body weight)
VI	Carrageenan + extract (100mg/kg body weight)
VII	Carrageenan + extract (150mg/kg body weight)
VIII	Carrageenan + extract (200mg/kg body weight)
IX	Carrageenan + extract (250mg/kg body weight)

DMSO = Dimethyl sulphoxide

$$\mathrm{Percentage}\;\mathrm{Abundance}=\frac{\text{Concentration of each Phytochemical}}{\text{Total Concentration of all Phytochemicals}}\times 100$$

Where, Vc is the Mean edema volume in the control group and Vt is the Mean edema volume in the treated group.

### Statistical data analysis

Raw data on inflammation was obtained, recorded and entered into a spreadsheet. Descriptive statistics were computed and the data expressed as mean± SEM. Inferential statistics were done using One-Way Analysis of Variance (ANOVA), followed by Tukey's post hoc test for pairwise comparison and separation of means. The anti-inflammatory activities of the two DCM plant extracts wre compared using the unpaired student t-test. The level of significance was set at 99.5% (p≤0.005).

## Results

### Quantitative phytochemical analysis of anti-inflammatory compounds of *E. globulus* and *S. didymobotrya*

The GC-MS analysis of the DCM leaf extract of *E. globulus* (Labill) revealed the presence of several bioactive chemical compounds that have potential anti-inflammatory activities (Table 2).

**Table 2:** Phytochemical composition of anti-inflammatory compounds of *E. globulus* and *S. didymobotrya*

Compound name	Chemical class	*E.globulus* Retention time (min)	% abundance	*S.didymobotrya* Retention time (min)	% abundance
Borneol	Mono terpenoids	14.12	0.90	-	-
α-terpineol	Mono terpenoids	14.57	1.06	-	-
Terpinolene	Terpenoids	12.80	0.93	12.82	4.66
Aromadendrene	Terpenoids	18.04	1.95	-	-
Camphene	Terpenoids	10.11	2.01	10.11	9.11
alpha-phellandrene	Terpenoids	11.26	12.73	11.26	3.24
Limonene	Terpenoids	11.71	10.44	11.73	24.90
Alpha-pinene	Terpenoids	9.78	5.73	9.80	5.47

### Anti-inflammatory effects of DCM leaf extracts of *E. globulus* (Labill) and *S. didymobotrya* (Fresenius) on carrageenan-induced inflammation in mice

Generally, the administered doses of the DCM leaf extracts of *E. globulus* (Labill) and *S. didymobotrya* reduced carrageenan-induced edema in Swiss albino mice. This was evidenced by decreases in the circumference of inflamed paw after administration of the extracts.

In the 1^st^ hour, the DCM leaf extract of *E. globulus* (Labill), at the five doses of 50, 100, 150, 200 and 250 mg/kg bw, reduced the inflamed paw in a dose-related manner by 0.85 %, 1.45 %, 1.41 %, 2.02 % and 2.27 % respectively (Table 3). However, the extract dose of 25mg/kg bw did not reduce inflammation (Table 3). At this hour, the anti-inflammatory activity of *E. globulus* extract at doses of 50, 100, 150, 200 and 250 mg/kg bw was statistically similar and comparable to the activity of diclofenac sodium as presented in Table 3 (p > 0.005).

**Table 3:** Anti-inflammatory effect of dichloromethane leaf extract of *E. globulus* (Labill) on carrageenan-induced inflammation in Swiss albino mice

Group	Treatment	Percentage change in paw circumference (mm)				

		0h	1h	2h	3h	4h
**Normal** **control**	DMSO (vehicle)	100.00±0.00	100.00±0.00^bc^ (0.00)	100.00±0.00^b^ (0.00)	100.00±0.00 ^b^ (0.00)	100.00±0.00 ^b^ (0.00)
**Negative** **control**	Carrageenan + DMSO only	100.00±0.00	103.06±0.44^a^ (-3.06)	106.65±0.42^a^ (-6.65)	108.18±0.36^a^ (-8.18)	109.11±0.39^a^ (-9.11)
**Positive** **control**	Carrageenan + Diclofenac (15mg/kg bw)	100.00±0.00	98.23±0.15^d^ (1.77)	93.57±0.23^f^ (6.43)	92.11±0.32^ef^ (7.89)	90.03±0.17^ef^ (9.97)
**DCM leaf** **extract**	Carrageenan+ 25mg/kg bw	100.00±0.00	100.81±0.50^b^ (-0.81)	100.15±0.31 ^b^ (-0.15)	98.72±0.20^b^ (1.28)	97.28±0.15^c^ (2.72)
	Carrageenan+50mg/kg bw	100.00±0.00	99.15±0.01c^d^ (0.85)	98.13±0.18^c^ (1.87)	96.78±0.17 ^c^ (3.22)	96.11±0.30^c^ (3.89)
	Carrageenan+100mg/kg bw	100.00±0.00	98.55±0.16c^d^ (1.45)	96.14±0.27^d^ (3.86)	94.20±0.34^d^ (5.80)	93.87±0.19^d^ (6.13)
	Carrageenan+150mg/kg bw	100.00±0.00	98.59±0.15^cd^ (1.41)	95.30±0.04^de^ (4.70)	92.65±0.17^e^ (7.35)	91.40±0.44^e^ (8.60)
	Carrageenan+200mg/kg bw	100.00±0.00	97.98±0.18^d^ (2.02)	94.10±0.22^ef^ (5.90)	91.30±0.18^ef^ (8.70)	90.06±0.32^ef^ (9.94)
	Carrageenan+250mg/kg bw	100.00±0.00	97.73±0.23 ^d^ (2.27)	93.48±0.20^f^ (6.52)	90.91±0.34^f^ (9.09)	89.10±0.19^f^ (10.90)

Descriptive statistics expressed as mean ± SEM for five mice in every group. Values that have different superscript letters are statistically significant along the same column by one-way ANOVA accompanied by Tukey's post hoc test (p≤ 0.005). The values in brackets represent % inflammation inhibition.

In the second hour, the *E. globulus* (Labill) extract, at the doses of 50, 100, 150, 200 and 250 mg/kg bw, reduced the paw edema in mice by 1.87%, 3.86%, 4.70%, 5.90% and 6.52% respectively (Table 3). However, at this hour, the *E. globulus* (Labill) extract dose of 25 mg/kg body weight exhibited no anti-inflammatory effects. There was a significant variation in the anti-inflammatory effect of *E. globulus* extract at the six dosages (p < 0.005; Table 3). However, the anti-inflammatory activity of diclofenac was not significantly different compared to that of DCM extract of *E. globulus* (Labill) at the dose levels of 200 and 250 mg/kg body weight as presented in Table 3 (p > 0.005).

In the 3^rd^ hour, the *E. globulus* extract doses of 25, 50, 100, 150, 200 and 250 mg/kg bw reduced the inflamed paw of animals by 1.28%, 3.22%, 5.80%, 7.35%, 8.70% and 9.09% respectively (Table 3). The anti-inflammatory effects of the six dose levels of *E. globulus* DCM leaf extract exhibited significant differences (p < 0.005; Table 3). However, the anti-inflammatory effect of the reference drug was comparable to those of the *E. globulus* extract doses of 150, 200 and 250 mg/kg bw as shown in Table 3 (p > 0.005).

In the 4^th^ hour, the *E. globulus* extract doses of 25, 50, 100, 150, 200 and 250 mg/kg bw decreased the inflamed paw circumference in mice models by 2.72%, 3.89%, 6.13%, 8.60%, 9.94% and 10.90% respectively (Table 3). There were significant variations in the anti-inflammatory effects of *E. globulus* extract at all the six tested doses in mice (p < 0.005; Table 3). However, the anti-inflammatory effect of diclofenac was statistically similar to those of the *E. globulus* extract doses of 150, 200 and 250 mg/kg bw (p > 0.005; Table 3). Generally, the *E. globulus* extract exhibited a dose-dependent response at all the hours tested (Table 3). Notably, the percentage paw circumference in the negative control group mice was significantly higher compared to those of extract-treated mice as well as those of rats in the positive control and normal control groups (p < 0.005; Table 3).

On the other hand, the DCM leaf extract of *S. didymobotrya*, at the six tested doses demonstrated anti-inflammatory effects on carrageenan-induced inflammation in Swiss albino mice (Table 4). In the first hour, the DCM extract of *S. didymobotrya* extract doses of 100, 150, 200 and 250 mg/kg bw decreased the inflamed paw circumference in mice by 1.11%, 1.40%, 2.14% and 2.41% respectively. However, the extract never exhibited anti-inflammatory effects at 25 and 50mg/kg body weight dose levels (Table 4). The anti-inflammatory activities of the *S. didymobotrya* extract doses of 100, 150, 200 and 250 mg/kg body weight exhibited no significant differences (p > 0.005; Table 4). Similarly, the anti-inflammatory activity of the reference drug (diclofenac) was comparable to those of *S. didymobotrya* extract doses of 150, 200 and 250mg/kg bw (Table 4; p > 0.005).

**Table 4:** Anti-inflammatory effect of dichloromethane leaf extract of *S. didymobotrya* (Fresenius) on carrageenan-induced inflammation in Swiss albino mice

Group	Treatment	Percentage change in paw circumference (mm)				

		0h	1h	2h	3h	4h
**Normal** **control**	DMSO (vehicle)	100.00±0.00	100.00±0.00^b^ (0.00)	100±0.00^b^ (0.00)	100.00±0.00^b^ (0.00)	100.00±0.00^b^ (0.00)
**Negative** **Control**	Carrageenan + DMSO	100.00±0.00	102.86±0.41^a^ (-2.86)	106.54±0.42^a^ (-6.54)	108.40±0.29^a^ (-8.40)	109.51±0.36^a^ (-9.51)
**Positive** **Control**	Carrageenan + (Diclofenac 15mg/kgbw)	100.00±0.00	97.27±0.0.21^e^ (2.73)	94.53±0.41^d^ (5.47)	92.95±0.21^f^ (7.05)	91.98±0.16^fg^ (8.02)
**DCM:leaf** **Extract**	Carrageenan+25 mg/kgbw	100.00±0.00	101.30±0.0.33^b^ (-1.30)	100.50±0.20^b^ (-0.50)	98.87±0.20^bc^ (1.13)	97.41±0.19^c^ (2.59)
	Carrageenan+50 mg/kgbw	100.00±0.00	100.50±0.20^b^ (-0.50)	99.50±0.20^b^ (0.50)	98.01±0.20^c^ (1.99)	96.84±0.16^c^ (3.16)
	Carrageenan+100 mg/kgbw	100.00±0.00	99.89±0.20^cd^ (1.11)	97.46±0.15^c^ (2.54)	96.19±0.29^d^ (3.81)	95.39±0.16^d^ (4.61)
	Carrageenan+150 mg/kgbw	100.00±0.00	98.60±0.15^de^ (1.40)	96.29±0.15^c^ (3.71)	94.89±0.21^de^ (5.11)	93.66±0.27^e^ (6.34)
	Carrageenan+200 mg/kgbw	100.00±0.00	97.86±0.16^de^ (2.14)	95.88±0.38^cd^ (4.12)	94.50±0.13^e^ (5.50)	92.97±0.24^ef^ (7.03)
	Carrageenan+250mg/kgbw	100.00±0.00	97.59±0.13^de^ (2.41)	94.57±0.27^d^ (5.43)	91.69±0.41^f^ (8.31)	90.95±0.32^g^ (9.05)

Descriptive statistics expressed as mean ± SEM for five mice in every group. Descriptive statistics that have different superscript letters are statistically significant along the same column by one-way analysis of variance accompanied by Tukey's post hoc test (p≤ 0.005). The values in brackets represent % inflammation inhibition.

In the 2^nd^ hour, the DCM leaf extract of *S. didymobotrya*, at doses of 50, 100, 150, 200 and 250 mg/kg bw, decreased the inflamed paw circumference in mice by 0.50%, 2.54%, 3.71%, 4.12% and 5.13% respectively (Table 4). However, the extract dose level of 25mg/kg body weight never revealed anti-inflammatory activity (Table 4). The anti-inflammatory activity of the tested doses of *S. didymobotrya* extract exhibited significant differences in mice (p < 0.005; Table 4). However, the anti-inflammatory activity of diclofenac was statistically insignificant compared to that of *S. didymobotrya* at the doses of 200 and 250 mg/kg bw (p > 0.005; Table 4). In the 3rd hour, the S. didymobotrya extract at doses of 25, 50, 100, 150, 200 and 250 mg/kg bw reduced the inflamed paw circumference by 1.13%, 1.99%, 3.81%, 5.11%, 5.50% and 8.31% respectively (Table 4). The anti-inflammatory effects of *S. didymobotrya* extract exhibited significant differences at the six tested doses at this hour (p<0.005; Table 4). However, the effect of the diclofenac was comparable to that of the DCM extract of *S. didymobotrya* at the dose level of 250mg/kg bw in Swiss albino mice (p > 0.005; Table 4).

The *S. didymobotrya* extract doses of 25, 50, 100, 150, 200 and 250 mg/kg body weight reduced the inflamed paw circumference by 2.59%, 3.16%, 4.61%, 6.34%, 7.03% and 9.05% respectively in the fourth hour (Table 4). At this hour, there was a significant variation in the anti-inflammatory effects of *S. didymobotrya* extract in mice at the six tested doses (p<0.005; Table 4). However, the anti-inflammatory effect of the reference drug, diclofenac, was comparable to that of extract at dose of 250mg/kg body weight (p >0.005; Table 4). Generally, the percentage paw circumference in the negative control group mice was significantly higher compared to extract-treated mice as well as mice in the positive and normal control groups (p < 0.005; Table 4).

### Comparison between anti-inflammatory activities of DCM extracts of *E. globulus* (Labill) and *S. didymobotrya* (Fresenius)

**Figure 2:**
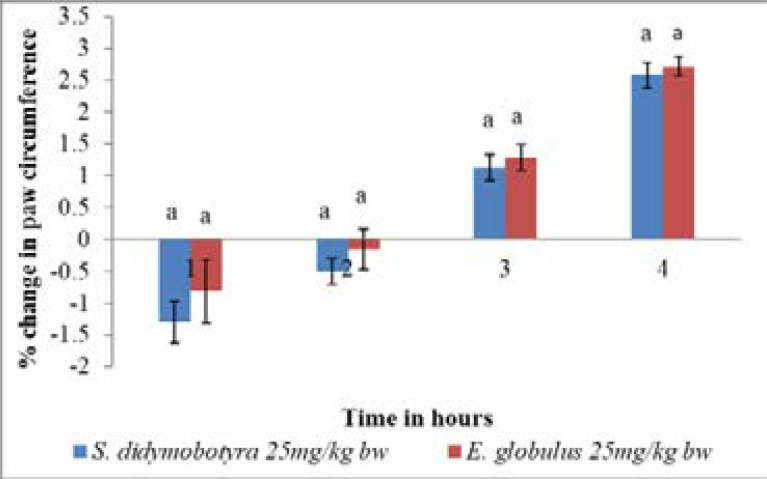
Comparison of anti-inflammatory activity of *E. globulus* (Labill) and *S. didymobotyra* (Fresenius) DCM leaf extracts at 25mg/kgbw on carrageenan-induced inflammation in mice. Means with different letters are statistically significant in each hour of treatment (p≤ 0.005). bw = bodyweight

In comparison, the anti-inflammatory activities of the DCM leaf extracts of the two plant extracts in mice, at the dose of 25mg/kg body weight, were not significantly different in the four hours of the test period (p > 0.005; Figure 2).

**Figure 3:**
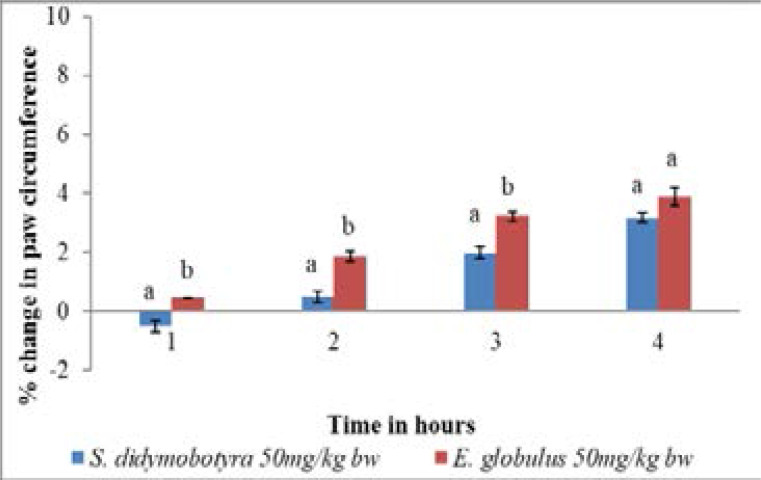
Comparison of anti-inflammatory activities of *E.globulus* (Labill) and *S. didymobotyra* (Fresenius) DCM leaf extracts at 50mg/kgbw dose level on carrageenan-induced inflammation in mice. Means with different letters are statistically significant in each hour of treatment (p≤ 0.005). bw = bodyweight

**Figure 4:**
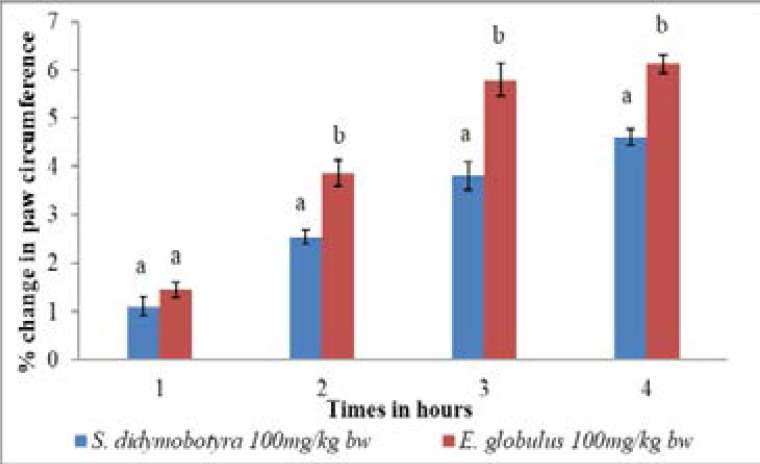
Comparison of anti-inflammatory effects of *E. globulus* (Labill) and *S. didymobotyra* (Fresenius) DCM leaf extracts at100mg/kgbw on carrageenan-induced inflammation in mice. Means with different letters are statistically significant in each hour of treatment (p≤ 0.005). bw = bodyweight.

Comparatively, the anti-inflammatory activity of the *E. globulus* extract dose level of 50mg/kg body weight was significantly higher compared to that of *S. didymobotyra* in the first, second and third hours at the same dose level (p<0.005; Figure 3). However, at the same dose level, the anti-inflammatory effects of *E. globulus* (Labill) and *S. didymobotyra* (Fresenius) extracts showed no significant differences in the fourth hour(p > 0.005; Figure 3). At the dose level of 100mg/kg body weight, the anti-inflammatory effects of DCM leaf extracts of *E. globulus* and *S. didymobotyra* were not significantly different in the first hour (p > 0.005; Figure 4). In contrast, the anti-inflammatory effect of *E. globulus* extract was significantly higher compared to that of *S. didymobotyra* at the same dose in the second, third and fourth hours (p≤0.005; Figure 4).

**Figure 5:**
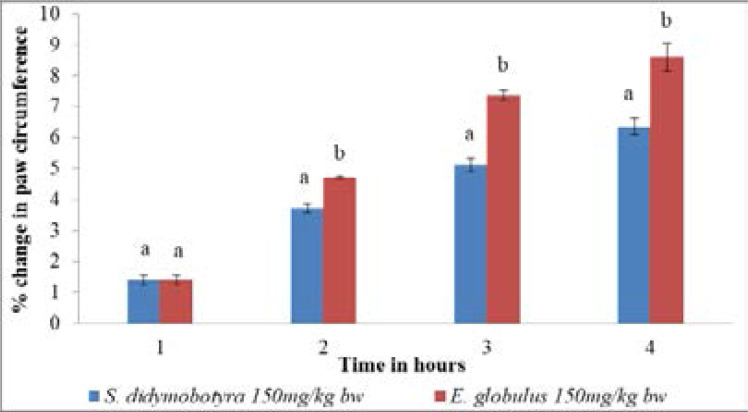
Comparison of anti-inflammatory effects of *E. globulus* (Labill) and *S. didymobotyra* (Fresenius) DCM leaf extracts at 150mg/kgbw on carrageenan-induced inflammation in mice. Means with different letters are statistically significant in each hour of treatment (p≤ 0.005). bw = bodyweight.

The anti-inflammatory effects of *E. globulus* and *S. didymobotyra* extract dose levels of 150mg/kg body weight revealed no significant difference in mice (p > 0.005; Figure 5). However, the anti-inflammatory activity of *E. globulus* extract at the same dose was significantly higher compared to that of *S. didymobotyra* in the second, third and fourth hours (p≤0.005; Figure 5).

**Figure 6:**
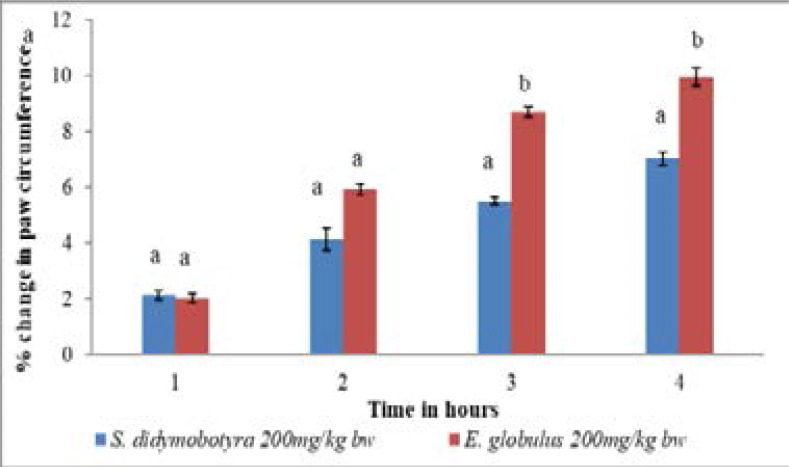
Comparison of anti-inflammatory effects of *E. globulus* (Labill) and *S. didymobotyra* (Fresenius) DCM leaf extracts at200mg/kgbw on carrageenan-induced inflammation in mice. Means with different letters are statistically significant in each hour of treatment (p≤ 0.005). bw = bodyweight.

There was insignificant variation in the anti-inflammatory effects of *E. globulus* and *S. didymobotyra* extract doses of 200mg/kg body weight in the first and second hours (p > 0.005; Figure 6). However, the anti-inflammatory effects of the *E. globlus* extract, at the same dose level, were significantly higher than that of *S. didymobotyra* in the third and fourth hours (p< 0.005; Figure 6).

**Figure 7:**
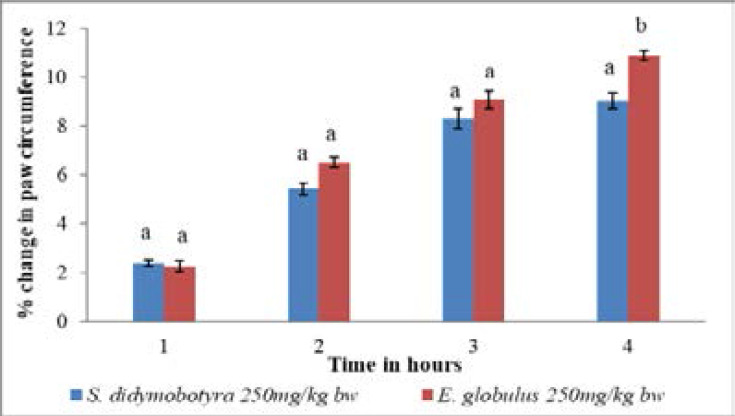
Comparison of anti-inflammatory effects of *E. globulus* (Labill) and *S. didymobotyra* (Fresenius) DCM leaf extracts at 250mg/kgbw on carrageenan-induced inflammation in mice. Means with different letters are statistically insignificant in each hour of treatment (p≤ 0.005). bw = bodyweight.

At the dose level of 250mg/kg body weight, the anti-inflammatory effects of DCM leaf extracts of *E. globulus* and *S. didymobotyra* in mice were statistically similar in the first, second and third hours (p > 0.005; Figure 7). However, in the fourth hour, there was an insignificant variation in the anti-inflammatory effects of the two plant extracts (p<0.005; Figure 7).

## Discussion

The present study was designed to determine anti-inflammatory potential of dichloromethane leaf extracts of *E. globulus* (Labill) and *S. didymobotrya* (Fresenius) in mice models. The studied plants are used by Mbeere community in Embu County, Kenya in the management of inflammation but no scientific research data is available to confirm their use.

Inflammation is a complex protective reaction of the body against noxious agents like microorganisms or injured cells^21^. Carrageenan-induced paw inflammation is the most suitable model in the determination of the anti-inflammatory activities of different agents that act by blocking the acute inflammatory mediators in vivo^22^. Carrageenan induces inflammation in two phases^23^. The early phase, which occurs between ninety to one hundred and eighty minutes of the inflammation, is as a result of the discharge of serotonin, histamine among other substances while the late, phase which occurs between 270 to 360 minutes, is postulated to be linked with the activation of the lysosome, prostaglandins and 62 proteases^24^.

The NSAIDs like diclofenac reduce edema by blocking enzyme cyclooxygenase (COX), which catalyzes prostaglandin biosynthesis^25^. Cyclooxygenase enzymes exist in two forms; cyclooxygenase-1 and cyclooxygenase-2. These two enzymes catalyze the synthesis of prostaglandins, which stimulate inflammation. The NSAIDs blocks the cyclooxygenase enzymes and decrease prostaglandin levels in the entire body, leading to a reduction of inflammation^26^, ^27^.

These study findings revealed that the *E. globulus* (Labill) and *S. didymobotyra* (Fresenius) DCM leaf extracts possess anti-inflammatory effects, which were evident by the reduction of inflamed paw circumference in mice models after treatment. These findings are consistent with earlier research works on the anti-inflammatory effects of other herbal plants in experimental animals. A study by Owolabi *et al.*^28^ on anti-inflammatory activity of aqueous, ethanol, diethyl ether, n-Hexane root extracts of *Feretia apodanthera* on carrageenan-induced paw edema in rats, reported that this extract provided great relief by lowering edema in the inflamed paw. A study by Matthew *et al.*^29^, who carried out studies on pharmacological activities of ethanol and aqueous extracts of dried stem of plant *Kalanchoe pinnata* (Lam.) Pers against inflammation in rats revealed potent anti-inflammatory activities.

The anti-inflammatory effects of these plants are attributed to their constituent phytochemicals like terpenoids, flavonoids and essential oils. Other researchers have associated these phytochemicals with anti-inflammatory activities. A review by Andrade and De Sousa^30^, demonstrated that α-Pinene possesses anti-inflammatory activity in mice. Another study by Bayala *et al.*^31^ revealed that essential oils possess anti-inflammatory activity in animal models. A study by Zou *et al.*^32^ revealed that borneol possesses significant anti-inflammatory activity. The results of GC-MS similarly revealed the presence of Alpha-terpineol. According to a study by Soleimani *et al.*^33^, Alpha-terpineol possesses anti-inflammatory activity in rat models.

The results of the GC-MS also revealed the presence of terpinolene. A study by Aggarwal *et al.*^34^, has shown that terpinolene isolated from turmeric has an anti-inflammatory effect on formalin-induced arthritis, cotton pellet granuloma and granuloma pouch models of inflammation in rats. The results of GC-MS analysis also revealed the presence of α-phellandrene. α-phellandrene is a monoterpene that is part of the essential oils found in many plants. Additionally, Gbenou *et al.*^35^, demonstrated that *Cymbopogon citratus* and *Eucalyptus citriodora* contained essential oils that possess significant anti-inflammatory activity by reducing formol-induced paw edema in rats. Similarly, a review by Arruda *et al.*^36^ also reports the anti-inflammatory potential of α-phellandrene. Further, the GC-MS results revealed the presence of Limonene. In a study by Li *et al.*^37^, on in vitro anti-inflammatory activity of *Spondias pinnata* (*Anacardiaceae*) on Nitric oxide production, indicated that Limonene possesses anti-inflammatory activity. Studies by Vieira *et al.*^38^, Rufino V.^39^, Khodabakhsh *et al.*^40^, also demonstrate that Limonene has great potential in the management of inflammation. According to a study by Jin *et al.*^41^, on the phytochemical composition of the cannabis plant and its use for medicinal purposes, it is reported that Aromadendrene and terpenoids possess anti-inflammatory effects.

## Limitations

This study focused on *in vivo* anti-inflammatory effects of *E. globulus* (Labill) and *S. didymobotrya* (Fresenius) in mice and did not explore *in vitro* anti-inflammatory activity of these plant extracts. Secondly, extracts of other plant parts like roots and stems were not studied for comparison purposes. Further, the extraction solvent used in this study was Dichloromethane, a non-polar solvent. Use of a polar extraction solvent may probably yield superior results.

## Conclusion

This study has demonstrated that the dichloromethane leaf extracts of *E. globulus* (Labill) and *S. didymobotrya* (Fresenius) have anti-inflammatory activity in mice, which can be attributed to the constituent phytochemicals. This study confirms the traditional claim of anti-inflammatory activities of the two plants.

## Abbreviations

ANOVA = Analysis of variance; DMSO = Dimethyl sulfoxide; ICIPE = International Centre of Insect Physiology and Ecology; GC-MS = Gas chromatography-Mass spectrophotometry; DCM = Dichloromethane; Bw = Body weight; kg = Kilogram; MS excel = Microsoft excel; NSAIDS = Non-Steroidal Anti-Inflammatory Drugs.
